# Oncologic and Functional Outcomes of Salvage Robot-Assisted Radical Prostatectomy: Report of the First 10 Cases

**DOI:** 10.3390/curroncol31080356

**Published:** 2024-08-20

**Authors:** Takahiro Oshina, Yuta Yamada, Tetsuya Fujimura, Satoru Taguchi, Yoshiyuki Akiyama, Jun Kamei, Tomoyuki Kaneko, Taketo Kawai, Daisuke Obinata, Daisuke Yamada, Hiroshi Fukuhara, Tohru Nakagawa, Satoru Takahashi, Haruki Kume

**Affiliations:** 1 Department of Urology, Graduate School of Medicine, The University of Tokyo, Tokyo 113-8655, Japan; oshinat-uro@h.u-tokyo.ac.jp (T.O.); satorutaguchi33@gmail.com (S.T.); ju.kmi27@gmail.com (J.K.); yamadai77777@gmail.com (D.Y.); kumeh-uro@h.u-tokyo.ac.jp (H.K.); 2 The Institute of Medical Science, The University of Tokyo, Tokyo 108-8639, Japan; 3 Department of Urology, Jichi Medical University, Tochigi 329-0498, Japan; tfujimura@jichi.ac.jp; 4 Department of Urology, Shinshu University School of Medicine, Nagano 390-8621, Japan; y.akiyama0208@gmail.com; 5 Department of Urology, Teikyo University School of Medicine, Tokyo 173-8606, Japan; kanekot@med.teikyo-u.ac.jp (T.K.); nakagawat@med.teikyo-u.ac.jp (T.N.); 6 Department of Urology, International University of Health and Welfare Ichikawa Hospital, Chiba 272-8501, Japan; taketokawai@yahoo.co.jp; 7 Department of Urology, Nihon University Itabashi Hospital, Tokyo 173-8610, Japan; obinata.daisuke@nihon-u.ac.jp (D.O.); takahashi.satoru@nihon-u.ac.jp (S.T.); 8 Department of Urology, Kyorin University School of Medicine, Tokyo 181-0004, Japan; hifukuha@gmail.com

**Keywords:** salvage radical prostatectomy, robot-assisted radical prostatectomy, prostatectomy, radiotherapy, focal therapy

## Abstract

Background: Salvage robot-assisted radical prostatectomy (sRARP) after PSA failure in patients who underwent initial radiotherapy or focal therapy has rarely been reported in Japan. We aimed to report the oncologic and functional outcomes of the first 10 cases of sRARP. Methods: Ten patients underwent sRARP after failing to respond to initial radiotherapy or focal therapy. Initial definitive treatment included volumetric modulated arc therapy, intensity-modulated radio therapy, stereotactic body radiotherapy, heavy-ion radiotherapy, low-dose-rate brachytherapy, and high-intensity focused ultrasound. We retrospectively investigated 10 cases on oncologic and functional outcomes of sRARP. Results: The median PSA level at sRARP, amount of blood loss, and console time were 2.17 ng/mL, 100 mL, and 136 min, respectively. Positive surgical margins were found in half of the cases. Median follow-up was 1.1 years. There were no 30-day major complications. No patients had erections after sRARP. Urinary continence and biochemical recurrence (BCR) rate were 40% and 30% at 1 year after sRARP, respectively. Conclusions: Salvage RARP may be a feasible option after PSA failure in patients who underwent radiotherapy or focal therapy as initial treatment, showing acceptable BCR rate.

## 1. Introduction

Prostate cancer is the most frequently diagnosed cancer among men in more than half of the countries in the world, with an estimated 1.4 million new cases in 2020 [1]. In the USA, 288,300 new cases of prostate cancer were newly diagnosed in 2023 [2]. This trend has been growing even in Asian countries, including Japan [3]. Robot-assisted radical prostatectomy (RARP) has now become a gold standard approach for treating organ-confined prostate cancer. However, salvage RARP (sRARP) after radiotherapy or focal therapy has rarely been reported in Japan. We experienced 10 cases of sRARP and report the oncologic and functional outcomes.

## 2. Materials and Methods

From September 2017 to May 2021, 10 patients underwent sRARP after PSA failure of initial radiotherapy or focal therapy. Six cases of sRARP were performed at the Tokyo University Hospital, three at the National Center for Global Health and Medicine, and one at Jichi Medical University. Clinical characteristics at the time of initial radiotherapy or focal therapy are shown in Table 1. Initial definitive treatment was volumetric modulated arc therapy (VMAT) in two cases, intensity-modulated radiotherapy (IMRT) with androgen deprivation therapy (ADT) in one case, stereotactic body radiotherapy (SBRT) in one case, heavy-ion radiotherapy (HIR) with ADT in two cases, low-dose-rate (LDR) brachytherapy in two cases, and high-intensity focused ultrasound (HIFU) in two cases. Biochemical recurrence (BCR) after initial treatment was defined based on the RTOG-ASTRO Phoenix Consensus Conference, i.e., prostate-specific antigen (PSA) increase of ≥2 ng/mL higher than the PSA nadir value, regardless of the nadir serum concentration [4]. Castration-resistant prostate cancer (CRPC) was defined as castrate serum level of testosterone (testosterone < 50 ng/dL) and three consecutive rises of PSA, 1 week apart, resulting in two 50% increases over the nadir with PSA > 2.0 ng/mL [5]. Notably, one patient with CRPC was included in the study. BCR after sRARP was defined as two postoperative PSA measurements ≥ 0.2 ng/mL [6].

All patients had biopsy and/or ^11^C-choline positron emission tomography (PET), ^18^F-fluorodeoxyglucose (FDG) PET, multiparametric magnetic resonance imaging (mpMRI) and/or computed tomography (CT) preoperatively to assess local presence of prostate cancer or metastatic lesions. Biopsy of the seminal vesicles were not performed before salvage RARP taking into consideration the dissemination of cancer cells. Clinical and pathological staging was performed according to the 2017 AJCC/UICC TNM classification system, eighth edition [7]. ‘Thirty-day complication’ was graded using the Clavien–Dindo classification [8,9]. Clavien–Dindo grade ≥ 3 was defined as major complications. Data on urinary continence and erectile function were collected at 1 year after sRARP. All procedures were performed through a six-port transperitoneal approach using the four-arm da Vinci Si or Xi^®^ Surgical Systems (Intuitive Surgical Inc., Sunny-Vale, CA, USA), as described in the previous study [10].

## 3. Results

Perioperative characteristics of sRARP are shown in Table 2, Tables S1 and S2. The median age at sRARP was 71 years (interquartile range (IQR): 66–79). The median time from initial treatment to sRARP was 89 months (IQR: 63–111). Six patients received ADT prior to sRARP (initial radiotherapy with ADT: two cases, initial radiotherapy with ADT and ADT after biochemical failure: one case, and ADT after biochemical failure: three cases). The median PSA level at sRARP was 2.17 ng/mL (IQR: 1.64–2.79). Cancer lesions were evident in the prostate, based on biopsy or imaging tests such as ^11^C-choline PET, in all cases (Table 2 and Table S1). Metastasis was not detected in any of the cases according to preoperative imaging tests (Table 2 and Table S1). Perioperative outcomes and complications of sRARP are shown in Table 2. The median estimated blood loss (EBL) and console time of sRARP were 100 mL (IQR: 50–100) and 136 min (IQR: 123–167), respectively. The median duration of catheterization was 7 days (IQR: 6–7). There were no major intraoperative or 30-day complications. Two patients required urethral dilation for anastomotic stricture at 9 months and 3 years after sRARP.

Oncologic and functional outcomes are shown in Table 3 and Table S2. Final pathological stage showed pT2 in six, pT3a in two, and pT3b in two cases. Five patients showed positive surgical margins, of which four cases were positive at the apex. All ten patients achieved PSA level of <0.2 ng/mL 1 month after salvage RARP. There were biochemical recurrences at 3, 7, and 8 months after sRARP in three cases. BCR-free survival rates at both 1 year and 3 years were 70%. Cancer-specific survival rates at 1 year and 3 years were both 100%. No patients had erections after sRARP. Four patients recovered urinary continence 1 year after sRARP.

Perioperative data and oncologic and functional outcomes compared with the previous reports are shown in Table 4 [11,12,13,14,15,16]. Median age, catheter placement, and potency rate in patients undergoing sRARP in previous studies ranged from 65 to 70 years, 10 to 16 days, and 5.2 to 27%, whereas in the present study, it was 71 years, 7 days, and 0%, respectively.

## 4. Discussion

Biochemical failure after non-surgical treatment in prostate cancer occurs in approximately 30% of the patients [17,18]. Most of the patients prefer ADT to sRARP as salvage therapy since sRARP is associated with severe complications, including urinary incontinence. A few studies on follow-up after sRARP have been published [11,12,13,14,15,16]. In the present study, we report the initial 10 cases of sRARP.

Compared with previous reports, duration of time to salvage RARP from initial treatment tended to be longer in the present study. This may have been due to the patient preference leaning on ADT treatment at the time of initial biochemical recurrence, resulting in a delay in the timing of salvage RARP. Age at sRARP in our study was also older than previous reports. This is based on our previous experience showing safety and tolerability in performing RARP in patients over 75 years of age [19]. All cases were successfully treated without major complications.

Compared with the previous studies, operative time and estimated blood loss tended to be shorter and less, probably owing to having done only one case of sparing procedure in the present study.

Complications included a slightly higher incidence of anastomotic strictures, but no serious complications such as rectal injury, vesicourethral anastomosis leakage, or cases with artificial urethral sphincter placement were observed. However, it should be noted that previous studies have observed rectal injury as a major complication after sRARP [11,12,16]. Thakker et al. raised a valuable suggestion in such a situation [20]. The pelvis should be filled with saline, and air should be instilled in the rectum through a rectal probe or a catheter to see any evident air bubbles [20]. In such cases, consultation with the general surgeon regarding colostomy or a two layered repair should be considered [20]. Nathan et al. compared oncologic and functional outcomes of sRARP with those of standard RARP [16]. The surgical outcomes of sRARP versus standard RARP were as follows: grade III–IV Clavien–Dindo complication rates: 1.5% vs. 0% (*p* = 0.310), pad-free continence rates: 78.8% vs. 84.3% at 2 years (*p* = 0.337), erectile dysfunction rates: 94.8% vs. 76.3% (*p* < 0.001) [16].

Although the positive surgical margin rate was relatively high, biochemical recurrence-free survival rate was consistent with other reports. In our study, only one patient had a positive margin that actually developed biochemical recurrence, although the follow-up period was relatively short. All cases showed remnant cancer at the time of sRARP, but it should be noted that all cases should have undergone prostate biopsy prior to sRARP. Presence of vascular invasion were observed in all cases with recurrence after sRARP. The PSA nadir value did not fall to the lower limit in two cases. Notably, in the recurrent cases, the median time from initial radiotherapy or focal therapy to sRARP tended to be much shorter, compared with recurrent-free cases (BCR cases: 57 months, BCR-free cases: 103 months).

The rate of achieving urinary continence and erection were significantly lower than other studies. Previous studies have shown favorable results for sRARP when focal therapy was implemented as initial therapy [16,17]. Accordingly, cases after HIFU were associated with good continence outcomes, as shown in cases No. 2 and No. 5 in the present study (Tables S1 and S2).

After initial radiotherapy, the endopelvic fascia is often thickened and fused to the prostate and levator ani muscles, and in some cases, the presence of dense fibrosis between Denonvilliers’ fascia and the prerectal fascia makes the development of posterior planes extremely complex [21]. Therefore, highly skilled and experienced surgeons should perform the salvage RARP and special attention must also be paid to avoid entering the incorrect dissection planes and causing injury to pelvic tissues.

There were several limitations within the present study. Firstly, this study was small-sample-sized and one cannot draw a decisive conclusion regarding treatment outcomes. However, it provides an accumulation of evidence regarding patients undergoing salvage RARP.

Another limitation was the lack of performing PSMA/PET CT. As this image test is not covered by insurance in Japan, it is not available in regular clinical practice and it was not performed in any of the patients included in this study. Under the circumstances where PSMA/PET CT is unavailable in regular clinical practice, there may be a debate as to whether PLND should have been performed in the nine cases of sRARP, which is also a major limitation. In this study, pelvic lymph node dissection was performed in only one case (case No. 9). In case No. 9, PLND during sRARP was performed since the authors were not completely sure regarding the presence of lymph node metastasis based on a CT scan. We performed PLND during salvage RARP and resected 68 lymph nodes, of which 1 lymph node turned out to be positive. Fortunately, this patient achieved a PSA level under 0.2 ng/mL 1 month after salvage RARP.

## 5. Conclusions

Salvage RARP provides oncologic control with an acceptable rate of complications. A longer follow-up is necessary to better assess the functional and oncologic outcomes.

## Figures and Tables

**Table 1:** Clinical characteristics at the time of initial radiotherapy or focal therapy (n = 10).

Median initial PSA, ng/mL (IQR)	11.0 (6.61–12.4)
Initial biopsy Gleason score, n (%)	
3 + 3	2 (20)
3 + 4	4 (40)
4 + 3	1 (10)
4 + 4	2 (20)
5 + 5	1 (10)
Initial definitive treatment, n (%)	
IMRT with VMAT	2 (20)
IMRT with ADT (2 years)	1 (10)
SBRT	1 (10)
HIR with ADT (2 years)	2 (10)
LDR brachytherapy	2 (20)
HIFU	2 (20)

IQR: interquartile range, IMRT: intensity-modulated radiotherapy, VMAT: volumetric modulated arc therapy, ADT: androgen deprivation therapy, SBRT: stereotactic body radio therapy, HIR: heavy-ion radiotherapy, LDR: low dose rate, HIFU: high-intensity focused ultrasound.

**Table 2:** Perioperative characteristics of sRARP (n = 10).

Median post-radiation PSA nadir, ng/mL (IQR)	0.65 (0.48–0.88)
History of ADT before sRARP *, n (%)	6 (60)
Median preoperative PSA, ng/mL (IQR)	2.17 (1.64–2.79)
Diagnosis of cancer at the prostate lesion prior to sRARP	
Re-biopsy of the prostate	7
Imaging test of the prostate (CT + MRI or ^11^C-choline PET)	3
Diagnosis of metastasis prior to sRARP	
CT + other imaging test (^11^C-choline PET, bone scintigram, ^18^F-FDG PET, DWIBS)	6
^11^C-choline PET	4
Median age at sRARP, years (IQR)	71 (66–79)
Median time since initial radiotherapy or focal therapy, months (IQR)	89 (63–111)
Median console time, min (IQR)	136 (123–167)
Median EBL, mL (IQR)	100 (50–100)
PLND, n (%)	1 (10)
Nerve-sparing, n (%)	1 (10)
Median catheterization time, days (IQR)	7 (6–7)
Median follow-up duration, months (IQR)	13 (10–26)
Major complication †, n (%)	
Intraoperative	0 (0)
30-day	0 (0)
Total	3 (30)
Anastomotic stricture	2 (20)
Refractory osteomyelitis	1 (10)

PET: positron emission tomography, MRI: magnetic resonance imaging, EBL: estimated blood loss, PLND: pelvic lymph node dissection, * initial radiotherapy with ADT: 2 cases, initial radiotherapy with ADT and ADT after biochemical failure: 1 case, ADT after biochemical failure: 3 cases. † Clavien–Dindo grade ≥ 3 was defined as major complication.

**Table 3:** Oncologic and functional outcomes after sRARP (n = 10)

Pathological T stage, n (%)	
T2	6 (60)
T3a	2 (20)
T3b	2 (20)
Pathological N+, n (%)	1 (10)
PSM, n (%)	5 (50)
Apex	3 (30)
Base	1 (10)
Apex and base	1 (10)
BCR, n (%)	3 (30)
Median time to sRARP since initial radiotherapy or focal therapy, months (IQR)	
in BCR cases	57 (45–81)
in BCR-free cases	103 (88–140)
Cancer-specific survivor, n (%)	10 (100)
Erection, n (%)	0 (0)
Pad free or safety pad *, n (%)	4 (40)
HIFU	2 (20)
HIR	1 (10)
LDR	1 (10)
Median time to urinary continence, months (IQR)	10 (9–10)

PSM: positive surgical margin, BCR: biochemical recurrence. * There was 1 patient with pad-free status (case No. 5) and 3 patients achieved safety-pad status (case No. 2, 3, and 8).

**Table 4:** Study descriptions of previous and current studies

Case Series	Kaffenberger [11]	Yuh [12]	Ogaya-Pinies [13]	Onol [14], §		Marconi [15]	Nathan [16]	Present Study
Year	2013	2014	2019	2019		2019	2021	2023
N.	34	51	96	94	32	82	135	10
Prior treatment (initial radiotherapy or focal therapy)	B, E, HF	B, E, P, HF Cr	B, E, HF, Cr, Cy	B, E, I, P	HF, Cr	F(A)	B, E, HF, Cr	B, I, S, HR, HF
Age (years)	67	68	66	65	66	65	70	71
Time to sRARP (months) *	48.5	68	81.5	82.2	61.1	NR	NR	89
Operative time (min)	176	179	125	129	122	NR	165	136
EBL (mL)	NR	175	100	107	93	400	200	100
Catheter placement (days)	NR	15	12	16	10	NR	NR	7
Continence rate (%) †	39 ‡	45	57.3	45.7 ‡	84.4 ‡	83	80.7	40
Potency rate (%) †	21 ‡	23	17.7	13 ‡	27 ‡	14	5.2 ‡	0
PSM (%)	26	31.4	16.7	17	43.8	13	37.8	50
BCR-free survival rate (%) †	82	57 ‡	84.4	64.9 ‡	68.8 ‡	74	68.1	70
Follow-up (months)	16	36	14	32	29	24	17	13

B: brachytherapy, E: external-beam radiotherapy, I: intensity-modulated radiotherapy, P: proton-beam therapy, Cr: cryotherapy, HF: high-intensity focused ultrasound, Cy: cyberknife, F: focal therapy, A: ablation, S: stereotactic body radiotherapy, HR: heavy-ion radiotherapy, NR: not reported, * time to sRARP since initial radiotherapy or focal therapy. † at 1 year after sRARP. ‡ at last follow-up. § this study including radiotherapy of 94 cases and focal ablation therapy of 32 cases.

## Data Availability

All relevant data are available from within the manuscript and Supplementary Materials.

## Supplementary Materials

The following supporting information can be downloaded at: https://www.mdpi.com/article/10.3390/curroncol31080356/s1, Table S1: All cases of salvage robot-assisted radical prostatectomy showing clinical characteristics, Table S2: All cases of salvage robot-assisted radical prostatectomy postoperative characteristics.
